# Modular composition predicts kinase/substrate interactions

**DOI:** 10.1186/1471-2105-11-349

**Published:** 2010-06-25

**Authors:** Yichuan Liu, Aydin Tozeren

**Affiliations:** 1 Center for Integrated Bioinformatics, Drexel University, 711 Bossone Research Building, 3120 Market Street, Philadelphia PA 19104, USA

## Abstract

**Background:**

Phosphorylation events direct the flow of signals and metabolites along cellular protein networks. Current annotations of kinase-substrate binding events are far from complete. In this study, we scanned the entire human protein sequences using the PROSITE domain annotation tool to identify patterns of domain composition in kinases and their substrates. We identified statistically enriched pairs of strings of domains (signature pairs) in kinase-substrate couples presented in the 2006 version of the PTM database.

**Results:**

The signature pairs enriched in kinase - substrate binding interactions turned out to be highly specific to kinase subtypes. The resulting list of signature pairs predicted kinase-substrate interactions in validation dataset not used in learning with high statistical accuracy.

**Conclusions:**

The method presented here produces predictions of protein phosphorylation events with high accuracy and mid-level coverage. Our method can be used in expanding the currently available drafts of cell signaling pathways and thus will be an important tool in the development of combination drug therapies targeting complex diseases.

## Background

Transient interactions of proteins with other proteins, such as those that occur during phosphorylation events, comprise a fundamental element of signal processing in living cells [1]. Protein kinases constitute one of the largest families of signaling proteins in eukaryotic cells [2]. Currently, there are more than 500 known protein kinases in the human genome [3]. A phosphorylated amino acid distinguishes itself from the unmodified residue by having a large hydrophilic group with increased hydrogen-bonding, hydration and salt-bridge formation capability. Such modifications often result in switches and altered lines of connections in signaling and metabolic pathways of living cells [1]. Phosphorylation binding interactions are important downstream in gene expression pathways in binding of transcription factors to their substrate proteins [4].

Transient interactions between proteins often require multiple sites of physical connection and may even require a third party protein such as an adopter protein. Catalytic phosphorylation events at active sites is facilitated either by the utilization of protein recognition modules or the adaptation of docking interactions [5]. Recent structural data indicates that specificity of binding between a kinase and a substrate does not necessarily arise from the active site but from substrate and the specific docking interactions [6]. Globular domain - motif interactions accompany active site interactions in the binding of tyrosine kinase to their substrates. Large numbers of such globular domain/linear motif interactions have already been associated with protein-protein interactions (PPI). Web tools such as PROSITE [7], Pfam [8], PRINTS [9], ProDom [10], and InterPro [11] can be used to annotate the globular domains and larger linear motifs on the sequence of any given protein. Similarly, the web tool ELM [12] annotates on protein sequences large numbers of linear motifs known to be involved in protein interactions. Some of these motifs may play important roles in virus-host interactions via a mechanism for hijacking function [13,14].

Known annotations of domain-motif interactions on protein partners often result in the prediction of large numbers of false positives in PPI [13]. It is also becoming clear that selectivity of docking sites in MAPK kinases along with the catalytic motif is an important player in identifying PPI [15]. An accurate method of PPI prediction based on interactions of short linear motifs on one protein with large globular domains on the protein pair is yet to be developed [16].

Computational prediction of PPI from primary sequences of proteins poses a number of other challenges to overcome including the noise in the training PPI data, lack of a true negative training set, as well as problems associated with 3D experimental and molecular modeling of proteins in potentially binding configurations [17]. PPI prediction methods that were developed in the last decade include methods based on sequence homology [18], feature vectors and machine learning methodology [19], association studies [20] and knowledge guided inference of domain-domain interactions from incomplete PPI networks [21]. Computational studies focusing on extracting domain signature pairs associated with PPI have utilized yeast datasets [20] or datasets spanning across species [21].

The success achieved in computational association of domain signature pairs with experimentally verified PPI in these aforementioned studies prompted us to investigate signature pair/PPI association in phosphorylation events within the human proteome. We asked the question whether modular composition of proteins (kinase and their substrates), combined with a database of known PPI, could be sufficient in a statistical enrichment procedure to predict known PPI not used in the training. The choice of domains as features for predicting PPI made sense because modular composition of proteins provides insights into their interaction with up and downstream proteins in cell signaling circuits [1,5].

In addressing this question, we used the Post Translational Modification (PTM) database 2006 edition containing 5602 PPIs to identify statistically enriched signature pairs in kinase/substrate binding. Our ten-to-one and two-to-one learning and testing procedures produced receiver operator characteristic curves reflecting excellent accuracy in the identification of phosphorylation events. Additional verification included the use of PPI in the PTM 2009 edition and in other databases not included in PTM [22-24]. Our bioinformatics analysis uncovered sets of domain clusters that are specifically enriched in various kinases and kinase substrates. Moreover, we showed that pairs of such domain clusters bridges kinase and kinase substrates with high specificity and sensitivity.

The computational space in our model is large compared to other approaches focusing only on the domain annotation of proteins known to be interacting with each other. In the present study we scanned the entire proteome for domain annotation in order to develop background sets of randomly generated virtual protein pairs to be used in statistical enrichment of domains in protein subsets. Another feature specific to our method is the consideration of strings of domains as signatures for binding predictions. This assumption facilitated us to consider binding events between proteins involving multiple sets of domains. Results produced by our method achieved better PPI prediction accuracy in phosphorylation on the average than other presently available computational methods for PPI. Our study illustrates the dominance of a grammar based on interacting domain signature pairs in the language of post modification interactions between proteins in the human proteome.

## Methods

### PPI data for phosphorylation events

The learning dataset on kinase/substrate binding was downloaded from the Post Translational Modification database (PTM), version 2006 [25]. The dataset contained 5602 phosphorylation events between 272 kinases and 1432 kinase substrates. The independent testing datasets consisted of phosphorylation events not recorded in PTM 2006 but recorded in the PTM 2009 [24], the Human Protein Reference Database (HPRD) [25], and the Biological General Repository for Interaction Datasets (BioGRID) [23]. Predictions of our model were used to match phosphorylated proteins in the PhosphoELM database [12] with candidate targeting kinases for further experimental verification.

### Scanning proteins for PROSITE domains and their enrichment in protein subgroups

Database of protein domains, families and functional sites named PROSITE [26] was downloaded to our Laboratory's Blade Center. In this set up, the search engine for PROSITE took protein FASTA sequences as inputs and returned hits of PROSITE domains (D) as outputs. Human protein sequences from the NCBI Gene Bank were scanned and a column matrix indicating the presence (1) and absence (0) of domains were assigned for each human protein. The dimension of these domain column matrices was equal to the number of domains (2102) in the PROSITE Database.

### Statistical enrichment of domains in protein subgroups

Statistical enrichment of domains in protein subgroups (target group) was performed with respect to control (background) group made of the entire protein kinase group. Domain column matrices were determined for each member of a target group and these matrices were summed up over the membership of the subgroup. Next, a set of proteins of the same number as the target group was selected randomly from the background group and the corresponding sum domain column matrix was computed. This operation was repeated 10,000 times and the p value for enrichment was computed by the fraction of times the background group had more domains of a given identity than the target group. List of domains (domain clusters) enriched in a kinase- or substrate subtype was identified as the list of signatures that are enriched in the target group, a kinase- or substrate subtype.

### Score matrix for signature pairs in PPI

A score matrix was constructed for selecting signature pairs strongly associated with known PPIs in tyrosine- and serine/threonine phosphorylation subgroups. Specifically we wanted to identify signature pairs (such as A-B) such that presence of signature A in protein K and signature B in protein L would predict with high confidence a PPI between K and L. For this purpose, for the known PPI interactions in the learning dataset (EPPI), we generated a score matrix whose rows and columns identified the enriched signatures in tyrosine and serine/threonine kinases (TK, S/TK) and their substrates (TKB, S/TKB). Each element of the matrix corresponded to the number of EPPI for which a signature pair (A-B) was present in the opposing proteins of the pair. Another score matrix for virtual PPI, VPPI (background), was generated by randomly pairing proteins from the learning dataset, in effect creating VPPI interactions equal in number to all possible protein combinations from kinase and substrate proteins in the PPI set. The p value for signature pair enrichment in a given PPI subgroup was computed using the hypergeometric test in the R Project for Statistical Computing, based on the scores summed from the learning set and the background set. The resulting signature pairs were ranked according to their p value, with the one corresponding to the lowest p value ranked highest. The highest p value used as cut-off in the analysis was p = 0.001.

### Prediction accuracy for string pairs

The signature pairs thus identified via statistical enrichment, were used to predict new PPI events. A protein pair was considered as undergoing phosphorylation interaction if they expressed at least one of the signature pairs determined by the enrichment analysis. Consider a protein pair (L, K) that is associated with a statistically enriched signature pair (A-B). Assumption that the presence of A-B means the presence of a phosphorylation PPI between L and K (PPPI) may lead to false positives. The prediction accuracy was evaluated by computing the probability that the match between predicted and experimental PPI sets to have occurred randomly. Consider there are *N *VPPI events that can be generated randomly from *n *kinases and *m *kinase substrates. Among the *N *VPPI, *M *have already been annotated as EPPI. Let the signature pair A-B predict *Y *number of PPPI, *W *of which have been verified as EPPI. The hypergeometric test than tests the probability of randomly choosing at least *W *EPPI by selecting Y PPPI out of a possible *N *VPPI. Lower the p value, higher is the accuracy of the PPI prediction method presented in this study.

### Sensitivity, specificity, precision, recall

In addition to p values, prediction accuracy was evaluated using parameters for defining accuracy and coverage: Specificity (*Sp*) and Sensitivity (*Se*). Let TP, TN, FP, and FN represent, respectively, the true positives, true negatives, false positives, and false negatives determined with the use of known PPI in the predicted set. *Sp *and *Se *were defined as follows:

The higher the value of *Sp*, the lower is the error for assuming PPI between L and K based on the presence of the enriched signature pair (A-B). Parameter *Se *is a measure of the coverage, namely the size of the PPI pool potentially predicted by A-B.

We also used precision and recall to evaluate the statistical enrichment of experimental PPI in our predicted PPI set. Precision (*Pr*) was defined as TP/(TP + FP). Recall (Re) is the same as the sensitivity parameter Se.

### Cross validation and validation with independent datasets

We used training and testing sets at 2-fold and 10-fold cross validation to test the accuracy of our predictions in 100 iterations using statistical enrichment with p values varying from zero to one [27]. After each set of training and testing we determined the specificity and sensitivity. We plotted the receiver operating characteristics (ROC) curve using the average values of specificity and sensitivity over 100 iterations. The area under the ROC curve (AUC) quantified the likelihood that one can identify a kinase-substrate interaction using the method described above.

In addition, we used multiple validation processes to evaluate the performance of our model. The first process was to check the accuracy of the enriched signature pairs in predicting PPI among the random protein pairs derived from proteins in the learning data set. A p value, representing the probability of randomly generated prediction was computed for the PPI predicted by each signature pair by using the hypergeometric test.

Next, we compared the PPI predictions based on PTM 2006 learning database with PPI not present in PTM 2006 but present in PTM 2009 and in two other databases (HPRD, BioGrid). We identified the phosphorylation PPI in the HPRD and BioGrid databases as those PPI made of a kinase and a substrate partner of the same type (tyrosine or serine/threonine) as listed in Gene Ontology [28]. For each comparison, we computed the number of PPI predicted, number of PPI matched, and the maximum number of virtual PPI that could be generated using the testing PPI dataset. These numbers yielded p values for random prediction using the hypergeometric test.

### Comparison with other computational models

We tested the accuracy of PPI predictions of the present model, with two previously published domain based methods: correlated sequence-signature markers (CSSM) [20] and the knowledge-guided inference of domain-domain interactions (K-GIDDI) [21]. Using the algorithms and data presented in these papers, we identified the enriched domain pair signatures and the resulting numbers of predicted PPI as well as the number of matched PPI (matching already annotated PPI) within the randomly generated PPI set from PTM 2006 as well as the validation datasets used for our model. We used the hypergeometric test, sensitivity, and specificity as described above to identify the accuracy of prediction.

## Results

### PROSITE domains enriched in kinase and their substrates

**Figure 1:**
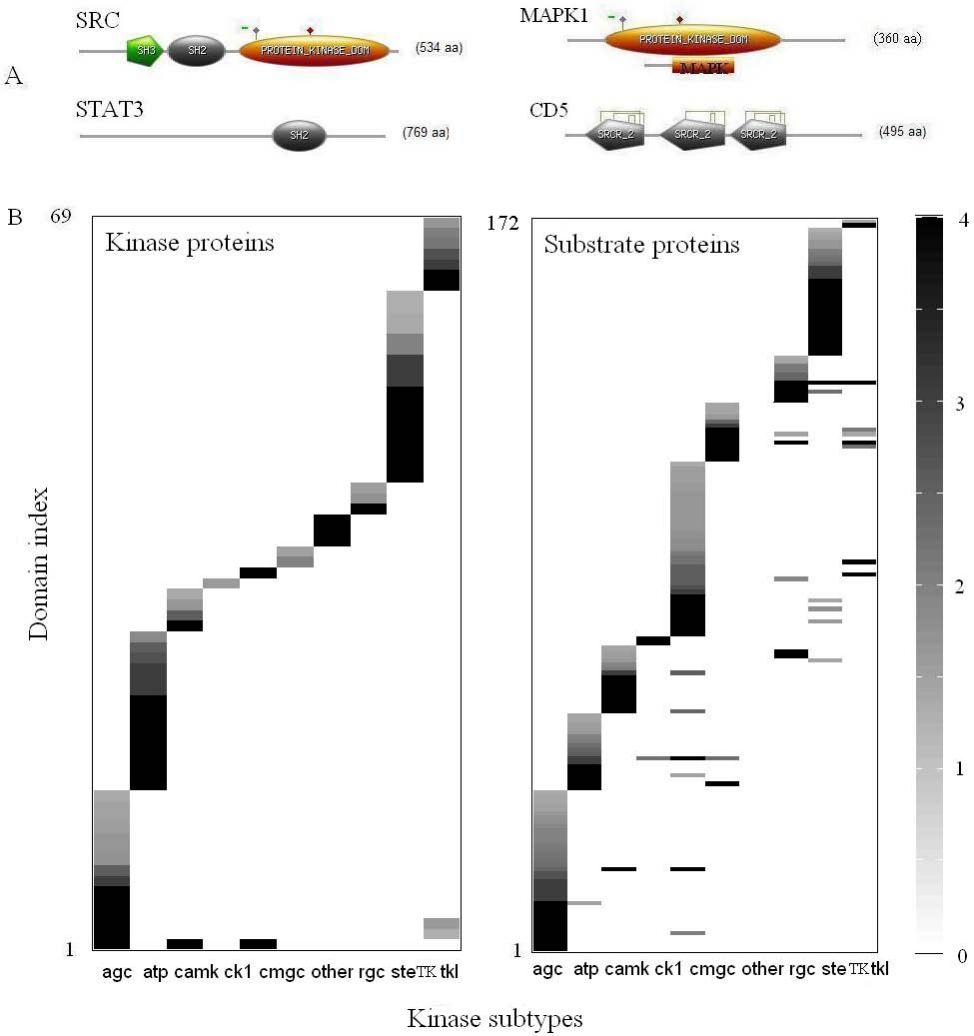
**Protein domains enriched in kinase- and substrate subtypes**. Example of protein domains annotated on the amino acid sequences of kinases and their substrates using PROSITE web tool screen shot (A), their statistical enrichment among kinase and substrate subtypes (B). The horizontal axis in B identifies the kinase (substrate) subtype (agc, atp, camk, ck1, cmgc, other, rgc, ste, TK, tkl) in the notation [2]. The vertical axes on the left refer to the identity index of statistically enriched domains in these subgroups of proteins (see Additional File 1 for key to the index). The scale on the right shows the - log p value of statistical enrichment of domains in these protein subgroups.

Our computations showed that kinases and their substrates express statistically enriched protein domains that are largely subtype specific. We scanned the human protein sequences in the NCBI database through the PROSITE web tool and identified the domains/signatures expressed on their sequences (Figure 1A). We then used statistical enrichment as described in the methods section to identify those domains enriched in a target kinase (substrate) subtype group against all kinase (kinase substrates) with enrichment p < 0.05. This enrichment procedure was carried out for the ten kinase subtypes described in Manning's paper. Figure 1B shows that domains enriched in a certain kinase (substrate) subtype are largely mutually exclusive to the subtype under consideration. The subtype specificity of domains expressed by kinase and substrates reduced drastically the number of domain signature pairs that needed to be considered for PPI prediction.

**Figure 2:**
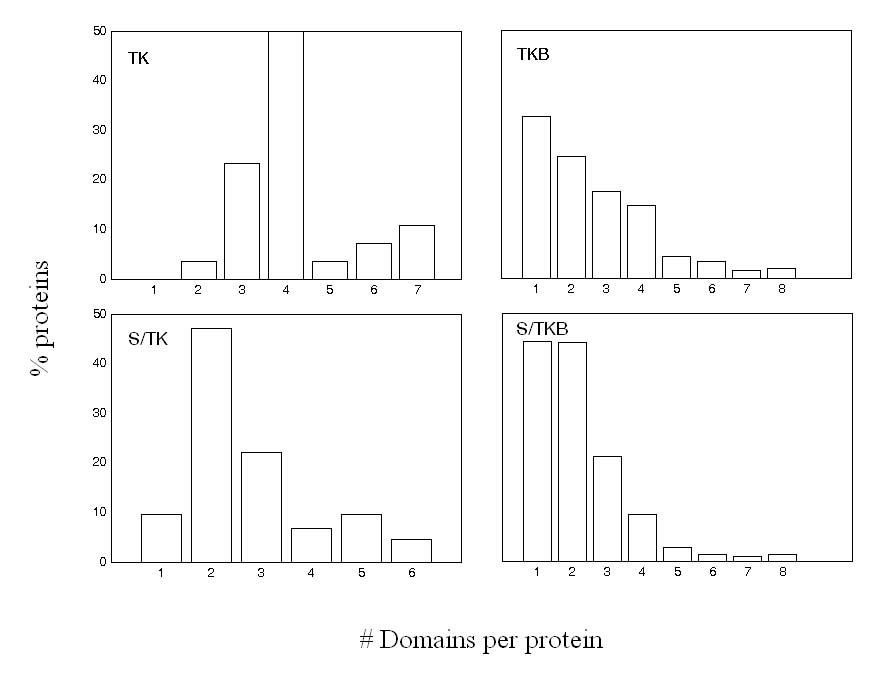
**Domain number density for kinases and their substrates**. Percentage of kinases (substrates) with N domains, N = 1, 2, 3,...,8. The symbols TK and S/TK identify tyrosine and serine threonine kinases, respectively. TKB and S/TKB are their substrates.

Next we considered the groups of enriched domains expressed by kinases and their substrates, grouped in two major subgroups: tyrosine and serine/threonine kinase (substrates). Many of these proteins expressed more than one subtype-specific enriched domain as shown in Figure 2. In other words, not only domains but domain strings were also enriched in tyrosine and serine/threonine kinase groups and their substrates. Therefore, each such enriched string of domains could be considered to constitute a signature. Additional File 1 contains the domains enriched in kinase and substrate subtypes along with the p values for enrichment. On the average, tyrosine kinase and their substrates have more protein domains than serine/threonine kinase and their substrates. This observation is consistent with the known preferred mode of interaction between tyrosine kinase and their substrates (domain-motif interactions) versus the docking site interactions employed in serine/theorine kinase [5].

### Score Matrices for identifying domain signature sets enriched in known kinase protein interactions

**Figure 3:**
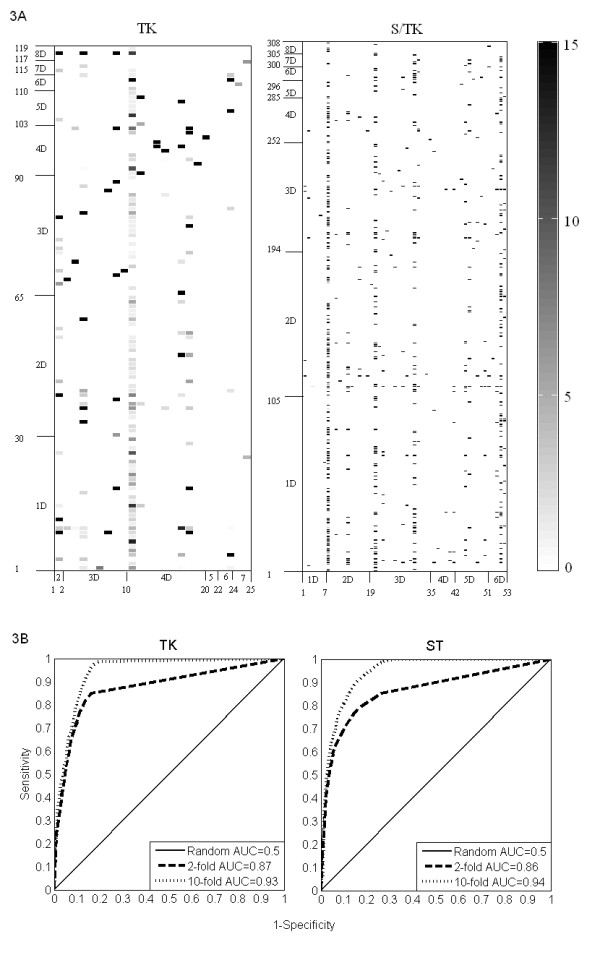
**Heat maps of the domain signature pairs associated with kinase-substrate interactions & ROC curves in cross-validation**. Pairs of strings of domains (one on a kinase, the other on its substrate) that are statistically enriched (p < 0.001) in phosphorylation binding interactions in the learning set compared to the set of random pairs of proteins made up of proteins in the same set. The horizontal and vertical axes refer to indices of strings of domains enriched in kinases and kinase substrates, respectively. The scale - log p indicates the level of enrichment of the signature pairs shown in the figure 3A. The receiver operating characteristics (ROC) curves for 2-folds and 10-folds cross-validation (3B). The area under the ROC curves (AUC) is also shown for the ROC curves in the figure.

A score matrix in our analysis has m rows and n columns with each row corresponding to one of the m enriched signatures (domains or string of domains) in a kinase category (TK or S/TK). Each column indicates one of the n enriched signatures in the corresponding substrate category (TKB or S/TKB) (Figure 3A). Elements of the target PPI score matrix show the number of times a signature pair is found in PPI in the PTM 2006 database. Elements of the virtual PPI score matrix show similarly the numbers of correlated signatures in this much larger pool of randomly generated protein pairs from the PTM 2006 proteins in PPI. Let M be the number of PPI under consideration and let N be the number of randomly generated protein pairs (including the actual PPI pairs), then hypergeometric test can be used to estimate the probability of a PPI score matrix element having the value *m *by chance when the corresponding value in virtual PPI score matrix is *n*. The negative logarithms of these p values for the correlated signature pairs are shown in Figure 3A on the score matrix heat maps for TK PPI (top) and S/TK PPI (bottom). Note that the smaller the p value, the darker is the matrix element corresponding to a signature pair. We have listed the identities of the signature pairs shown enriched in Figure 3A as Additional File 2. The file contains the pairs of signatures (in terms of PROSITE domain identity), their identity number along the horizontal axis of Figure 2, and the p value of enrichment.

The signature pairs presented in additional file 2 predicted nearly 80 percent of the PPI used in identifying the correlated signature pairs. Note that on the average each signature pair is correlated with ten PPI, suggesting that domain compositions of proteins involved in phosphorylation are indicative of their potential for binding. The p value shown in Table 1 training part for this case indicates the efficiency of our score matrix approach in correlating signature pairs with phosphorylation PPI events.

**Table 1:** Accuracy and coverage of the present approach for predicting kinase - substrate interactions

		Training				Testing			
		
		PTM	2006	HPRD		BioGrid		PTM	2009
		
		TK	S/TK	TK	S/TK	TK	S/TK	TK	S/TK
Data	EPPI	886	2925	137	199	111	166	33	237
	Kinase	56	176	60	104	41	67	15	61
	Substrate	274	881	32	44	26	44	21	90
	VPPI	15344	155056	1920	4576	1066	2948	315	5490

Present	PPPI	1132	7133	43	204	36	69	27	193
	MPPI	617	1876	18	26	17	15	6	16
	p value	0	0	9.0 E-12	1.3E-07	1.0E-09	7.0E-07	0.014	0.0038
	Se	69.6	64.1	13.14	13.07	15.32	0.04	18.18	6.75
	Sp	96.6	95.4	97.76	95.54	96.62	97.66	91.43	96.48

CSSM	PPPI	14496	122975	763	1411	433	836	234	4462
	MPPI	826	2815	84	67	64	53	26	160
	p value	0.9416	0	3.79E-08	0.0096	4.33E-05	0.128	0.204	1
	Se	93.23	96.24	61.31	33.67	57.66	31.93	78.79	67.51
	Sp	5.44	20.69	60.26	69.17	59.38	71.64	25.71	18.72

K-GIDDI	PPPI	1491	1557	117	75	88	68	33	65
	MPPI	206	42	19	6	17	5	2	11
	p value	0	0.0098	1.32E-04	0.0432	0.0025	0.1814	0.7	0.0002
	Se	23.25	1.44	13.87	3.02	15.32	3.01	6.06	4.64
	Sp	90.28	99	93.9	98.36	91.74	97.69	89.52	98.81

EPPI: Number of Experimental PPI; PPPI: Number of predicted PPI; MPPI: Number of matches between PPPI and EPPPI; VPPI: Number of Virtual PPI used in p value computations. Also shown is the prediction coverage and accuracy of two previously published approaches (CSSM, K-GIDDI).

### Cross validation and additional validation with independent experimental datasets

Approximately 75 percent of known kinase-substrate interactions occurred between proteins with at least one annotated PROSITE domain on their primary sequence. For cross validation, we used the kinase-substrate pair list in PTM 2006 and took its subset made of protein couples with both proteins expressing at least one annotated PROSITE domain. This restriction was necessary since our prediction method is based on existence of certain domain pairs (signature pairs) in interacting proteins. As described in the methods section, we used 10-fold and 2-fold cross validation in 100 iterations and generated receiver operating characteristic (ROC) curves for predicting tyrosine kinase and serine/threonine kinase interactions (Figure 3B). The figure indicates excellent accuracy at 10-fold cross validation and slightly lower accuracy in 2-fold cross validation. The areas under the ROC curves (AUC) for these cases are reported in the figure.

Next we compared our predicted PPI set with those phosphorylation PPI sets that have not been used in our statistical enrichment processes. Three PPI databases, BioGrid, HPRD, and PTM 2009, have contained hundreds of kinase/substrate phosphorylation events as shown in Table 1 testing part. We used the signature pairs listed in additional file 2 to predict PPI events among the proteins in the PPI events shown in Table1 testing set. The p values for the match between our predictions and the known PPI events not used in our enrichment procedures ranged from 9*10 ^-12 ^to 7*10 ^-7 ^for PPI presented in HPRD and BioGrid database whereas we had higher but still significant p values when predicting PTM 2009.

Next we compared the experimental data shown in Table 1 with the corresponding predictions that could be made using the domain based methods recently published (CSSM & K-GIDDI). These comparisons yielded p values that were larger than the ones for our method. In particular, the p values showed no significance for model CSSM predicting PTM 2009 and the serine/threonine binding data from BioGrid. The reason why our model yielded better results than CSSM could be due to our grammar differentiating between proteins with different domain string expression. Another reason maybe our use of randomly generated background PPI databases in our enrichment method rather than an analytical equation based only on data for PPI. Note also that CSSM model was for the yeast proteome and we used not their published results but generated PPI predictions using their procedure here for comparison with experimental data for the human proteome.

K-GIDDI simulation also yielded in higher p values than our method when compared with the human protein interactive data shown in Table 1. The comparison maybe unfavorable to K-GIDDI since the model incorporates PPI events from multiple species during training phase and therefore might miss some PPI events specific to human. Nevertheless, the fact that all these three approaches gave statistically significant predictions for at least the HPRD database indicates the validity of domain based approaches in predicting phosphorylation events. Sensitivity and specificity parameters were also computed for three approaches across different dataset. Present study shows same accuracy level of specificity as K-GIDDI and better coverage, CSSM show much better coverage but much less specificity.

Overall, our approach predicts 8837 kinase-substrate interactions from a pool of 186,715 virtual interactions and matches 2591 PPI out of the experimentally verified 4694 PPI. The p value for the match is zero and precision and recall are equal to 0.293 and 0.552, respectively. Predictions for tyrosine kinase mediated phosphorylation PPI is better in terms of precision than those PPI involving serine-theronine kinases (Table 2), but, nevertheless, both predictions match experimental data with zero p value for random match.

**Table 2:** Efficiency of the present score matrix enrichment in matching known phosphorylation PPI

	PPPI	EPPI	MPPI	VPPI	Precision	Recall	p value
Overall	8837	4694	2591	186715	29.3	55.2	0

TK	1238	1167	658	18645	53.2	56.4	0

S/TK	7599	3527	1933	168070	25.4	54.8	0

### Matching kinase with substrates in expanding previously annotated cellular pathways

**Figure 4:**
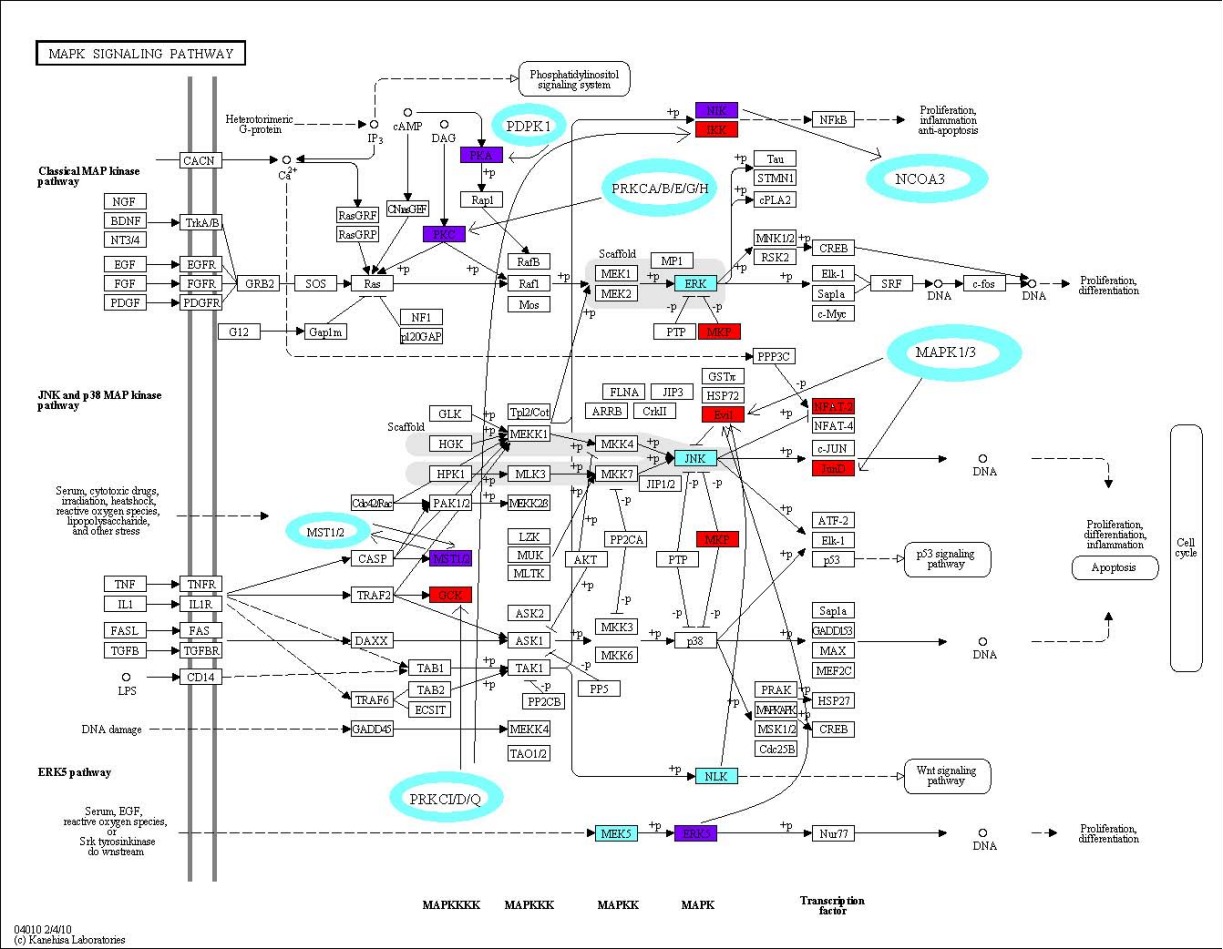
**KEGG MAPK Pathway revised by adding predicted phosphorylation events**. Brackets lined with red identify those nodes in the existing KEGG diagram occupied by a kinase substrate whereas the blue lined brackets identify those occupied by kinases. The proteins that act both as kinases and substrates are shown in purple. The oval shaped nodes are our predictions of kinases that also phosphorylate existing nodes in the KEGG diagram.

Nearly 30 percent of our predictions match experimentally verified phosphorylation PPI. We screened the substrates in the remaining 70 percent for their presence in the PhosphoELM [12] database. We found that an additional 30 percent of our predictions involved kinase substrates for which kinase partners are yet to be identified. for this reason, we wanted to see if our PPI prediction method could be used to revise and possibly expand previously annotated cellular pathways involved in signaling. Consider, for example, KEGG MAPK signaling pathway [29] showing a chain of phosphorylation events starting at the cell surface concluding with transcription factors that interact with DNA. A large number of the nodes in the figure are kinase substrates and our DDI based predictions of the corresponding kinase matches with those in the KEGG pathway (Figure 4). Nodes marked in red in the pathway are listed in PhosphoELM [12] as kinase substrates with unknown kinase identity. Our predicted kinase for those nodes has been added to the KEGG diagram. Out of the 11 predicted kinase/substrate interactions added to the KEGG pathway, 6 appear in HPRD or BioGrid databases, indicating that any expansions to previously established protein interactomes using our approach will likely be biologically relevant.

## Discussion

Binding interactions of proteins with other proteins are at the foundations of cellular networks. Phosphorylation is responsible for the flow of signals and metabolites along the protein pathways [17]. Dynamic binding interactions, such as those that occur in phosphorylation events, appear prominently in signaling pathways in health and in disease including hypertension, diabetes, HIV infection, and cancer [2,30]. Although kinases have long been considered as drug targets, compounds targeting kinases (kinase inhibitors and natural substances) have been found to be more promiscuous than originally anticipated, which can potentially lead to side effects [31]. It is important to identify potential phosphorylation partners of kinases in order to assess its range of impact on the flow of signals and metabolites along cellular pathways. Recent methods of mapping dynamic protein interactions in kinase signaling using live-cell fluorescence fluctuation spectroscopy and imaging already produced promising results [32] and kinase morphisms have been directly linked to population subtypes in disease states [33]. These new experimental approaches will benefit from the ongoing efforts in predicting dynamic protein interactions based on existing data and learning/testing/validation approaches. Our study produces this type of computational prediction sets of protein-protein interactions for experimental validation.

We used large-scale bioinformatics databases and tools and developed a methodology for predicting phosphorylation binding events that are yet to be fully annotated. Our method benefits from the hypothesis and assumptions of the previous computational methods of PPI prediction and specifically utilizes the concept of correlated sequence signatures as markers of protein-protein interaction developed by Sprinzak and Margalit (2001). The two new elements in our approach consist of (a) expanding the definition of signature to strings of domains rather than a single domain and (b) the use of background composed of random pairing of kinase and substrates in the statistical processes for identifying signature pairs indicative of phosphorylation events. The first assumption is consistent with our observation that certain strings of domains are highly statistically enriched in kinase subtypes and their substrates compared to the rest of the kinase interactome. The second assumption, requirement of statistical enrichment, against highly differentiating background sets, allowed us to further reduce the set of correlated sequence signatures obtained solely on the data involving PPI. The list of signature pairs developed in the present study, when used in predicting kinase/substrate interactions in phosphorylation events, produced results that are largely matched with experimental data not used in statistical enrichments for signature identification. The p values associated with our predictions and their comparison to independent experimental data ranged from a low of 10 ^-11 ^to 0.0038, depending on the kinase subtype and the database used for comparison.

Thousands of human proteins have been identified as undergoing phosphorylation binding interactions in the PhosphoELM database but the identity of the kinases responsible for these phosphorylation events are yet to be quantified. Our method produced candidate kinases targeting these substrates. The resulting list turned out to be consistent with literature not yet included into the PhosphoELM database. In all cases, the partnering between the substrate and the kinase predicted in this study can serve as a guide for kinase identification studies involving known kinase substrates. Another important use of our method will be in expanding and revising existing literature on cellular pathways decorated with phosphorylation events. Such revisions will be useful in identifying the consequences of small drug interventions on a kinase in terms of its interaction with immediate neighbors. Last but not least, our observation that domains expressed by kinase proteins and their substrates are largely subtype-specific drastically reduces the upper bound for the number of experiments one has to conduct for quantifying a major subset of transient binding interactions between protein pairs associated with phosphorylation.

One important disadvantage of our method is the bias toward the discovery of PPI with proteins having similar domain composition. This feature is also persistent in PPI prediction methods based on sequence homology. This tendency is observable in our prediction of new results included in PTM 2009 based on the PTM 2006 dataset. Although our match is statistically significant, the p values we get for this comparison is significantly larger than comparison with HPRD and BioGrid. It is expected that our methodology will pick up more PPI events correctly as we learn more about the protein sequence grammar that relates domain expression with protein-protein interaction patterns.

## Conclusions

Protein phosphorylation events redirect and redistribute the flow of signals and metabolites in cellular pathways. Kinases that phosphorylate multiple substrates have been favorable targets for drug development against many disease types. In this study, we developed a high throughput method that predicts potential binding partners for kinases using existing domain annotation tools and interactome databases for the human proteome. The method, when tested against independent databases, yields predictions with high statistical accuracy. Results indicate that domains expressed by any two proteins constitute a strong determinant of the potential for phosphorylation related binding interactions between them. Our expansion of the MAPK pathway using the prediction method outlined in the study presented results compatible with research literature.

## Authors' contributions

YL and AT conceived and developed the research plan. YL implemented the integration of algorithms and performed the computations. All authors worked on and approved the final manuscript.

## Supplementary Material

Additional file 1 **PROSITE domains that are statistically enriched in subtypes of kinases and substrates**. Columns of the table represent domain index used in Figure 1B, domain name, domain PROSITE ID Number as well as the kinase groups for which the domain is enriched. Click here for file

Additional file 2 **The list of domain-strings pairs used in predicting phosphorylation PPI with high specificity (SP > 0.91)**. DSIK: Domain string index for the kinase in PPI; DSIS: Domain string index for the substrate in PPI. Click here for file
